# Comparative Genomics of Multidrug Resistance in *Acinetobacter baumannii*

**DOI:** 10.1371/journal.pgen.0020007

**Published:** 2006-01-13

**Authors:** Pierre-Edouard Fournier, David Vallenet, Valérie Barbe, Stéphane Audic, Hiroyuki Ogata, Laurent Poirel, Hervé Richet, Catherine Robert, Sophie Mangenot, Chantal Abergel, Patrice Nordmann, Jean Weissenbach, Didier Raoult, Jean-Michel Claverie

**Affiliations:** 1 Information Génomique et Structurale, Institute for Structural Biology and Microbiology, IBSM, Marseille, France; 2 Génoscope, Centre National de Séquençage and CNRS UMR8030, Evry, France; 3 Département de Bactériologie-Virologie, Hôpital de Bicêtre, Le-Kremlin-Bicêtre, France; 4 Unité des Rickettsies, CNRS UMR6020, Faculté de Médecine, Université de la Méditerranée, Marseille, France; INSERM U571, France

## Abstract

*Acinetobacter baumannii* is a species of nonfermentative gram-negative bacteria commonly found in water and soil. This organism was susceptible to most antibiotics in the 1970s. It has now become a major cause of hospital-acquired infections worldwide due to its remarkable propensity to rapidly acquire resistance determinants to a wide range of antibacterial agents. Here we use a comparative genomic approach to identify the complete repertoire of resistance genes exhibited by the multidrug-resistant *A. baumannii* strain AYE, which is epidemic in France, as well as to investigate the mechanisms of their acquisition by comparison with the fully susceptible *A. baumannii* strain SDF, which is associated with human body lice. The assembly of the whole shotgun genome sequences of the strains AYE and SDF gave an estimated size of 3.9 and 3.2 Mb, respectively. *A. baumannii* strain AYE exhibits an 86-kb genomic region termed a resistance island—the largest identified to date—in which 45 resistance genes are clustered. At the homologous location, the SDF strain exhibits a 20 kb-genomic island flanked by transposases but devoid of resistance markers. Such a switching genomic structure might be a hotspot that could explain the rapid acquisition of resistance markers under antimicrobial pressure. Sequence similarity and phylogenetic analyses confirm that most of the resistance genes found in the *A. baumannii* strain AYE have been recently acquired from bacteria of the genera *Pseudomonas, Salmonella,* or *Escherichia*. This study also resulted in the discovery of 19 new putative resistance genes. Whole-genome sequencing appears to be a fast and efficient approach to the exhaustive identification of resistance genes in epidemic infectious agents of clinical significance.

## Synopsis

The bacterial species *Acinetobacter baumannii* is a major cause of hospital-acquired infection throughout the world, and it is an increasing public health concern due to its increasing resistance to antibiotic treatment. Coincidently, a high incidence of multidrug-resistant *A. baumannii* bloodstream infections was recently reported in US Army service members injured during Afghanistan and Iraq/Kuwait military operations. *A. baumannii* exhibits a remarkable ability to rapidly develop antibiotic resistance, which led from fully susceptible to multidrug-resistant strains within three decades. The authors used whole-genome sequencing and bioinformatic analyses to identify the complete repertoire of resistance genes exhibited by the multidrug-resistant *A. baumannii* strain AYE, which is epidemic in France, and to investigate the mechanisms of their acquisition by comparison with the fully susceptible *A. baumannii* strain SDF, which is associated with human body lice. This study led to the discovery in the AYE genome of an 86-kb region called a resistance “island”—the largest identified to date—that contains a cluster of 45 resistance genes. The homologous location in the susceptible strain, curiously, exhibited a 20-kb genomic island that is devoid of resistance markers. This ability to “switch” its genomic structure probably explains the unmatched speed at which *A. baumannii* captures resistance markers when under antibacterial pressure, such as is found in hospital intensive care units.

## Introduction

The prevalence of nosocomial infections in hospital intensive care units due to *Acinetobacter baumannii* currently ranges from 2% to 10% of all gram-negative bacterial infections in Europe [1] and account for about 2.5% of them in the United States [2]. *A. baumannii* exhibits a remarkable ability to rapidly develop antibiotic resistance that led to multidrug resistance (MDR) within a few decades [3]. To date, some strains of *A. baumannii* have become resistant to almost all currently available antibacterial agents [4], mostly through the acquisition of plasmids [5], transposons [6], or integrons [7,8] carrying clusters of genes encoding resistance to several antibiotic families [6–8] at once. With the emergence of increasingly resistant strains, the management of *A. baumannii* infections has become a public health problem in many countries. Recently, a high incidence of MDR *A. baumannii* bloodstream infections in US Army service members injured during Afghanistan and Iraq/Kuwait military operations was reported [9]. To date, no study has been designed to investigate at once the various resistance mechanisms involved in the acquisition of an MDR phenotype by a given *A. baumannii* strain. We had the opportunity to cultivate the MDR *A. baumannii* strain AYE, which is resistant to β-lactams (except imipenem, piperacillin-tazobactam, and ticarcillin-clavulanate), aminoglycosides, fluoroquinolones, chloramphenicol, tetracycline, and rifampin; is epidemic in 54 healthcare facilities in eight French administrative regions; and is associated with a mortality of 26% of infected patients [8]. Simultaneously, we also cultured the remarkably susceptible strain SDF, which is associated with human body lice [10]. We used a whole-genome sequencing approach to compare the gene content of *A. baumannii* AYE and SDF strains, with a special emphasis on gene categories related to antibacterial resistance. The parallel genome sequence annotation of both strains allowed us to identify all genes associated with previously known antibiotic resistance, and, unexpectedly, to discover that most of these genes were clustered in an 86-kb region, or “island,” of the *A. baumannii* strain AYE genome. To our knowledge, this is the largest resistance island described to date. In addition, we discovered 19 putative resistance genes not previously described in *A. baumannii*. The sequence analysis of *A. baumannii* AYE antibacterial resistance genes indicates that frequent genetic exchanges take place with *Pseudomonas* spp., *Salmonella* spp., or *Escherichia* spp.

## Results

### Shotgun Genome Sequences of *A. baumannii* Strains AYE and SDF

A preliminary assembly of the whole shotgun genome sequences of the strains AYE and SDF, as described below in Materials and Methods, led to a size estimate of 3.9 Mb and 3.2 Mb, respectively, with G + C contents of 38.8% and 38.2%.

In addition to their main chromosomes, the AYE and SDF strains harbor three plasmids (5, 9, and 94 kb) and two plasmids (6 and 25 kb), respectively. Surprisingly, none of the plasmids carry any known resistance marker. A detailed analysis of these two genome sequences will be published elsewhere; the present report focuses on the genes predicted to be involved in antibacterial resistance that, as expected, were found to account for the main difference in gene complement between *A. baumannii* strains AYE and SDF. These genes, together with the type of antibacterial resistance they display, are listed in Tables 1–3.

**Table 1:**
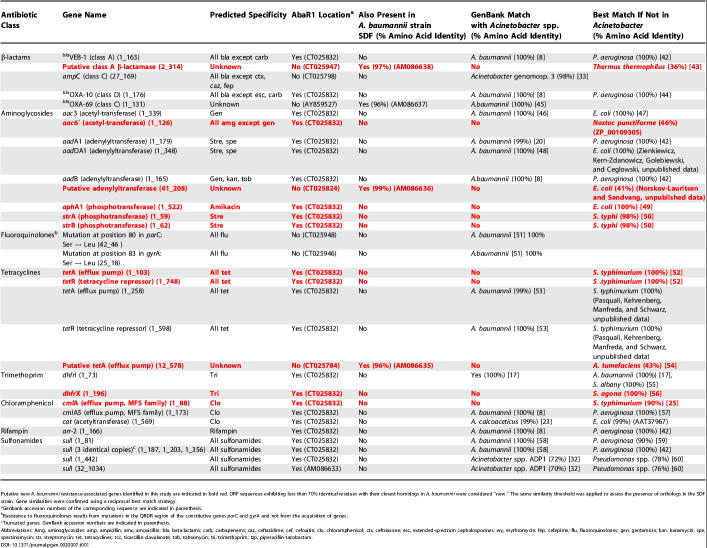
*A. baumannii* AYE Strain ORFs Putatively Associated with Resistance to Antibiotics

**Table 2:**
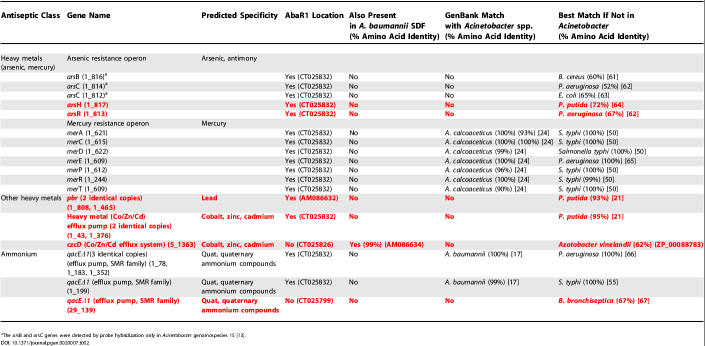
*A. baumannii* AYE Strain ORFs Putatively Associated with Resistance to Various Antiseptics

**Table 3:**
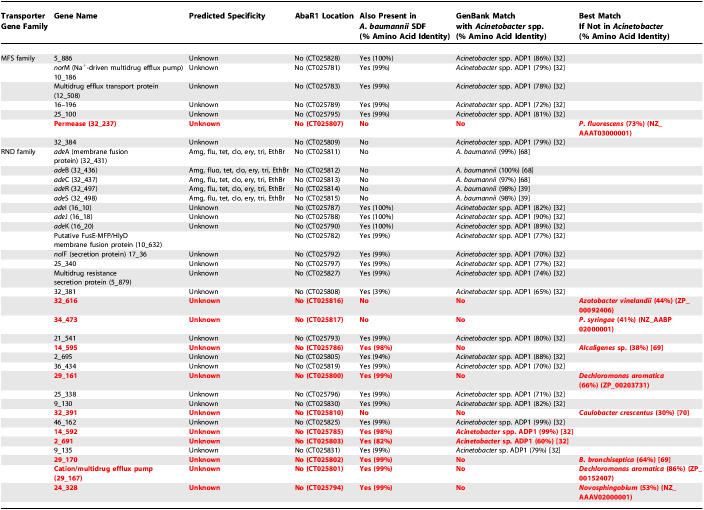
*A. baumannii* AYE Strain ORFs Encoding Putative Drug Transporters

**Table 3:**
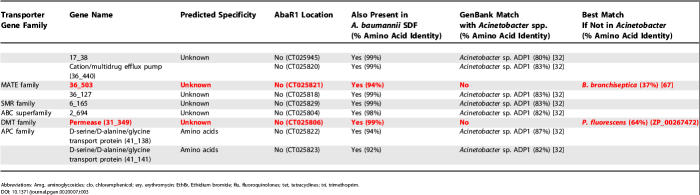
Continued.

### An 86-kb Resistance Island in *A. baumannii* Strain AYE

**Figure 1:**
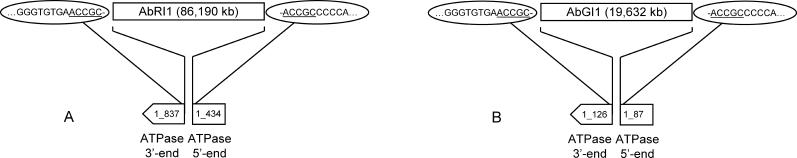
Comparison of Insertion Sites of the AbaR1 and AbaG1 Islands in an ATPase ORF Insertion sites of the AbaR1 (A) and AbaG1 (B) islands in an interrupted ATPase-encoding ORF are compared. ORFs 1_837 and 1_434 in strain AYE, and 1_126 and 1_87 in strain SDF, retain a strong similarity to the 3′ and 5′ fragments, respectively, of the ATPase sequence encoded by *Acinetobacter* strain ADP1 [32]. The 5-bp direct repeats flanking the islands are underlined.

**Figure 2:**
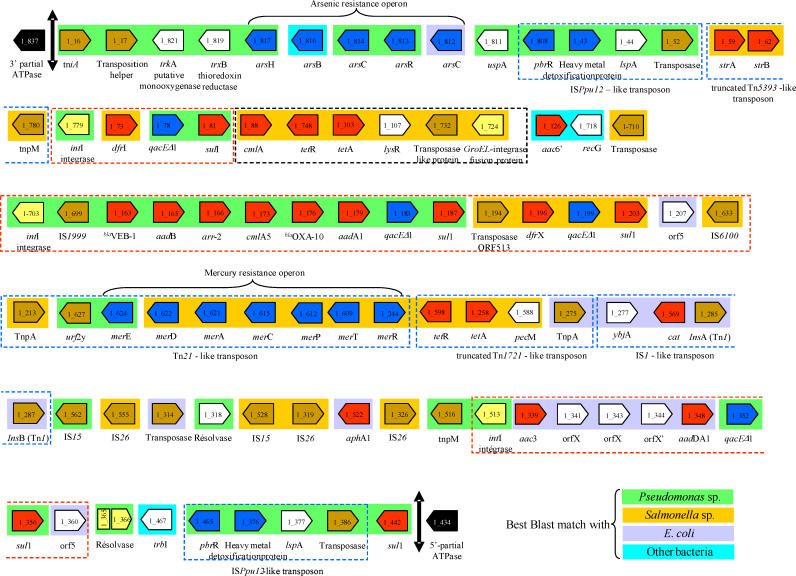
Layout of the Complete AbaR1 Inserted into the AYE Strain ATPase-Encoding Gene The nomenclature of each ORF is indicated. Each ORF is identified according to EMBL entry CT025832. Colors are used to indicate ORF categories: resistance to antibiotics in red, resistance to heavy metals or antiseptics in blue, transposases in brown, integrases in yellow, and other functions in white. Genes exhibiting a best matching homolog in *Pseudomonas* are underlined in green, in yellow for *Salmonella,* in light blue for *E. coli,* and in turquoise for other bacteria. Complete integrons are indicated by red dashed lines. Transposons are indicated by blue dashed lines. Black dashed lines indicate a gene cluster found in *Salmonella* genomic island 1.

**Figure 3:**
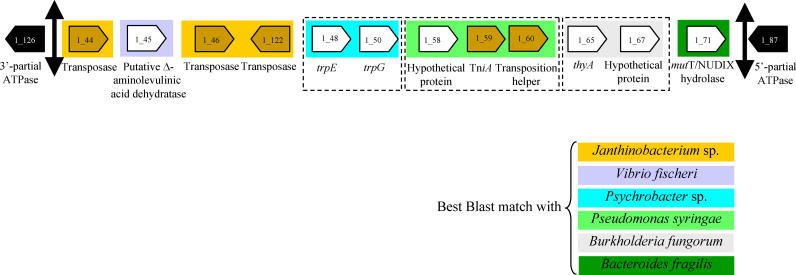
Layout of the Complete AbaG1 Inserted into the SDF Strain ATPase-Encoding Gene The nomenclature of each ORF is indicated. Each ORF is identified according to EMBL entry CT025833. ORF categories are indicated by colors: transpositions in brown and other functions in white. The phylogenetic origin of genes is highlighted by color markers. Dashed lines indicate genes found in the same order in other bacteria.

The AYE strain genome encoded 52 genes predicted to be associated with resistance to antimicrobial drugs, while only seven were identified in the SDF strain genome. Interestingly, 45 (86.5%) of the 52 AYE resistance genes are tightly clustered in an 86,190-bp genomic region disrupting a putative ATPase open reading frame (ORF) (Figures 1 and 2). The insertion site was identical in the two strains (Figure 1) and the insert was flanked at both extremities by a five-nucleotide direct repeat (ACCGC). This repetition is likely due to a duplication, which suggests a transposition mechanism for the insertion. However, no inverted repeats characteristic of transposons were found at the extremities of the island. The 86-kb region was classified as a genomic island (and designated AbaR1) on the basis of its size (>10 kb), marked G + C content difference from the rest of the chromosomes (52.8% versus 38.8%), the presence of genes associated with genome instability such as integrases, transposases, and insertion sequences, and the diverse phylogenetic origin of the associated ORFs [11]. At the same position, flanked by identical nucleotide sequences and within the homologous ATPase ORF, the *A. baumannii* strain SDF genome sequence exhibits a genomic island (designated AbaG1), albeit only 19,632 bp in length, with a G + C content of 31.3% (Figure 1). This shorter genomic island encodes 25 putative ORFs, 12 of which had a match in databases, but only ten of which were assigned a function: four transposases, a transposition helper, a thymidylate synthase, anthranilate synthase components 1 and 2, a putative Δ-aminolevulinic acid dehydratase, and a *mutT*/NUDIX hydrolase (Figure 3). Detailed sequence comparison of the two islands failed to reveal any significant similarity, including among the transposases found at their extremities.

Genomic islands containing resistance markers are referred to as resistance islands [12]. Resistance islands have been described mainly in γ-proteobacteria, including *Shigella flexneri, Salmonella enterica,* and *Vibrio cholerae,* but also in *Staphylococcus aureus* [11]. Their sizes range from 20 to 60 kb [11]. The AbaR1 resistance island specific to the *A. baumannii* AYE strain is thus, at 86 kb, to our knowledge the largest described to date, encoding 88 predicted ORFs, 82 of which could be assigned a predicted function (Figure 2, Tables 1–3). According to amino acid sequence similarities greater than 90% in most cases, 39 genes (44%) are likely to have originated from *Pseudomonas* spp., 30 (34%) from *Salmonella* spp., 15 (17%) from *Escherichia* spp., and four (4%) from other microorganisms. Of the 45 resistance genes, 25 are associated with various classes of antibiotics. These include genes that had not been previously described in *Acinetobacter* species: *str*A, *str*B, *aph*A1, and *aac*6′ (resistance to aminoglycosides); putative tetracycline-resistance genes *tet*A (tetracycline efflux pump) and *tet*R (repressor); *dfr*X (resistance to cotrimoxazole); and the chloramphenicol-resistance gene *cml*A (chloramphenicol efflux pump). In addition, genes previously found in *Acinetobacter* species were identified, including those that encode the β-lactamases VEB-1 and OXA-10; the aminoglycoside acetyl transferase gene *aac*3 and the aminoglycoside adenylyltransferases *aad*A1/DA1/B; the cotrimoxazole resistance-associated *dfr*I; *tet*A and *tet*R; *cml*A5 and one copy of the chloramphenicol acetyl-transferase *cat;* the rifampin ADP-ribosyltransferase gene *arr*-2; and five copies of the sulfonamide-resistance gene *sul*I encoding dihydropteroate synthetase, a component of class 1 integrons. The annotated sequences of the AbaR1 and AbaG1 islands, which include the genes discussed above, have been deposited in the EMBL database.

### Antiseptic Resistance-Associated Genes in *A. baumannii* Strain AYE

The AbaR1 also encodes two complete operons, one associated with arsenic resistance [13] and the second with mercury resistance (Figure 2) [14]. The latter is widely distributed both in clinical strains of gram-negative bacteria and in environmental bacterial strains [15]. The arsenic-resistance operon lacks the *ars*A and *ars*D genes [16] associated with high levels of resistance to both arsenic and antimony, but it includes *ars*H and *ars*R, which have not been found previously in *Acinetobacter* species. In addition, two genes encoding heavy metal efflux pumps, and four *qacEΔ1* genes encoding small multidrug resistance (SMR)-family efflux pumps, known to confer a low level of resistance to ammonium antiseptics, are scattered throughout the AbaR1 resistance island. The *qacEΔ1* genes were probably not acquired independently, as they are parts of the structure of class 1 integrons.

### Fine Structure of the AbaR1 Resistance Island

Fourteen of the antibiotic resistance genes could be mapped to three class 1 integrons (Figure 2). One integron carries *dfr*I [17]. A second integron is a composite made of a complete class 1 integron identified in *P. aeruginosa* [8], which carries *^bla^*VEB-1, *^bla^*OXA-10, *arr-*2, *cml*A, *aad*A1 [18], and a duplication of the 3′-conserved segment region that includes orf513 and *dfr*X cassettes. Such additional genes have been observed in *In*6-like class 1 integrons [19]. The third integron encodes *aac*3 and *aad*DA1 [20]. No other integron was detected outside of AbaR1 in the AYE strain genome, nor in the SDF strain genome.

In addition to integrons, a record 22 ORFs encoding transposases or other mobility-associated proteins were identified in the AbaR1 resistance island, suggesting that transposons are central to the island dynamic and the rapid acquisition of foreign resistance genes by the *A. baumannii* AYE strain. For instance, the island exhibits a near perfect duplication of a cluster of four genes including *pbr*R (a heavy metal-associated cation efflux transporter-encoding gene), *lsp*A (lipoprotein signal peptidase gene), and a gene encoding a tnpA-like transposase (Figure 2). Such a gene cluster was previously encountered in a *Pseudomonas putida* transposable element (IS*Ppu12*) [21]. Another four transposons, previously described in *Acinetobacter* spp. or other bacteria, were found within AbR1. These included a truncated Tn*5393* transposon made of *str*A, *str*B, and tnpM [22]; a truncated Tn*1721* transposon comprising *tet*R, *tet*A, *pec*M, and tnpA [22]; an IS*1*-like transposable element including *ybj*A, *cat, ins*A, and *ins*B [23]; and a Tn*21*-like transposon made of tnpA, *urf*2y, and the mercury-resistance operon [24]. Another five insertion sequences from the IS*15* and IS*26* classes were found in AbR1, including two IS*26* that flanked the *aph*A1 gene. In addition, the remaining five genes involved in antibiotic resistance were located near a transposase gene, thus suggesting a possible role of transposition in their acquisition.

Genomic islands are unstable regions and hotspots for the successive integration of resistance determinants in *Salmonella* spp., *Escherichia coli,* or *Streptococcus thermophilus* [12]. We can safely assume that the mosaic-like structure of the AbaR1 island is the result of successive acquisitions of DNA fragments from different hosts [12], mainly *Pseudomonas* spp., *Salmonella* spp., and *E. coli* (Figure 2). We also identified a cluster of genes similar to a fragment of the *Salmonella* genomic island 1 that comprises *cml*A, *tet*R, *tet*A, *lys*R, a transposase-like gene, and a *GroEL*-integrase fusion protein-encoding gene [25]. However, no obvious mechanism for these integrations is suggested by the structure of the *A. baumannii* genome. Although AbaR1 contains many genes found on plasmids in other bacteria, it is devoid of plasmid markers. It has been proposed that a strain of *A. calcoaceticus* could stably integrate antibiotic resistance genes carried by an R plasmid into its chromosome, and then discard the plasmid [6]. Such a mechanism could have occurred in the *A. baumannii* AYE strain. Another possible acquisition mechanism might involve natural transformation [26]. Transformation has been demonstrated in *Acinetobacter* spp. [27–31]. As a matter of fact, the first genome of an *Acinetobacter* spp. to be sequenced was that of the highly transformable *Acinetobacter* spp. strain ADP1 [32]. The close evolutionary proximity of *Acinetobacter* spp. and *Pseudomonas* spp. may facilitate direct gene exchanges between them.

### Antibacterial Resistance Genes outside of the Genomic Islands

In addition to the two β-lactamase genes located in AbaR1, three others were identified in AYE: the previously described *amp*C [33,34], surprisingly absent from the SDF strain, and two new putative β-lactamases (Table 1), both of which had orthologs in the SDF strain. One of them, a class D-type oxacillinase named *^bla^*OXA-69, was demonstrated to have a narrow-spectrum hydrolysis profile including, at low level, imipenem and meropenem [35]. We also identified an ORF encoding a 43-kDa porin that is homologous to the *Acinetobacter* heat-modifiable protein A [36]. The reduction in the expression of this porin, in concordance with the presence of an OXA-24 oxacillinase, is thought to play a role in carbapenem resistance in *A*. *baumannii* [37]. However, no ortholog to OXA-24 has been found in the AYE strain, and the level of expression of the putative porin is unknown.

Additional ORFs encoding a putative aminoglycoside adenylyltransferase and a tetracycline efflux pump *tet*A were also found in both *A. baumannii* strains. As these putative resistance genes did not confer a resistant phenotype to the SDF strain, their exact function is not known. In contrast, only the AYE strain exhibited a Ser → Leu mutation at positions 83 and 80 of the *gyr*A and *par*C genes, respectively, that confer resistance to fluoroquinolones [38].

An additional 46 ORFs putatively associated with resistance to antimicrobials were identified in the AYE strain. These ORFs encoded putative efflux pumps from the resistance-nodulation-cell division (RND) family (32 ORFs), major facilitator superfamily (MFS) family (seven ORFs), multidrug and toxic efflux (MATE) family (two ORFs), SMR family (one ORF), ATP binding cassette (ABC) superfamily (one gene), drug/metabolite transporter (DMT) family (one gene), and amino acids, polyamines, organic cations (APC) transporter family (two genes) (Table 3). Among the RND gene family, the AYE strain, but not the SDF strain, exhibited the AdeABC efflux pump-encoding genes (*ade*A, *ade*B, and *ade*C) and their regulating genes *ade*R and *ade*S. However, the latter two genes did not exhibit the Thr153 → Met or Pro116 → Leu mutations previously associated with an MDR phenotype [39]. Finally, both strains AYE and SDF possess the adeIJK efflux pump (encoded by *ade*I, *ade*J, and *ade*K).

Most of the genes associated with competence in *Acinetobacter* strain ADP1 [31,32,40] were identified in the sequences of the AYE and SDF strains (to be described elsewhere). However, as we have no direct evidence of natural transformation in these isolates, and as a single missing gene may kill the function, it is not yet certain that these genes play a significant role in the acquisition of new resistance genes.

## Discussion

This study demonstrates the usefulness of comparative genome sequencing for a rapid survey of all putative resistance mechanisms in *A. baumannii.* The determination and detailed comparison of the genome sequences of the MDR strain AYE, and the strain SDF, which is free from most resistance acquired by *A. baumannii* over recent decades, allowed the identification of many genes associated with antibacterial resistance at once. We identified 52 genes associated with resistance in the AYE strain, including 17 genes not, to our knowledge, previously described in *A. baumannii*. The clustering of 45 (86.5%) of them within an 86-kb AbaR1 resistance island was unexpected.

The detailed sequence analysis of the AbaR1 island, the largest genomic island identified to date, showed that it was built through the recursive insertion of broad host-range mobile genetic elements (transposons, gene cassettes from class 1 integrons), with gene cassettes (sometimes chimeric) mostly originating from the genera *Pseudomonas, Salmonella,* and *Escherichia*. Together with *A. baumannii,* many members of these genera are commonly found in aqueous environments of healthcare facilities, where, under antimicrobial pressure in these settings, genetic exchange among them may be promoted.

Another unexpected finding was the presence of a similar structure in the genome of susceptible strain SDF, identically inserted in the homologous ATPase-like ORF. This genomic island was found in an “empty” state, exhibiting mobility-associated genes but no resistance markers. This coincidental genetic insertion in the two strains strongly suggests that this ATPase ORF constitutes a specific hotspot of genomic instability in the *A. baumannii* genome. This prompted us to investigate whether this feature was common to all *A. baumannii* strains. Using a polymerase chain reaction assay based on the conserved ATPase ORF flanking sequences (Protocol S1), 17 (77%) out of the 22 clinical *A. baumannii* isolates were found to exhibit an intact ATPase ORF. These 17 isolates included 11 isolates resistant to several antibiotic families, including β-lactams, and six susceptible to β-lactams. Among the five isolates exhibiting an interrupted ATPase ORF, four were resistant to most β-lactams except imipenem, including some that were also resistant to other antibiotic families, and one was susceptible to β-lactams but resistant to cotrimoxazole and rifampin (Protocol S1). The presence of a genomic island within the ATPase ORF is thus not a conserved feature among isolates, and its absence at this location is not predictive of the observed pattern of antibiotic susceptibility. This suggests a particular flexibility of the *A. baumannii* genome, in line with its exceptional ability in gathering foreign genetic material. Whole-genome sequencing of additional *A. baumannii* strains will be needed to assess the full range of mechanisms through which clinical isolates can so efficiently acquire new resistance genes.

Finally, our last surprise was the identification of several putative resistance genes in a strain not exhibiting the associated phenotype. This was the case for two putative β-lactamases (present in the AYE and SDF), including the class D β-lactamase *^bla^*OXA-69. The expression of OXA-69 in *E. coli* resulted in low levels of carbapenem resistance [35], and its sequence is 97% identical to the OXA-51 carbapenemase, found associated with full carbapenem resistance [41]. However, the presence of *^bla^*OXA-69 in the susceptible AYE strain suggests that it might only be a step away from acquiring this new resistance through subtle changes in expression level or a specific mutation or series of mutations that alter the substrate profile and enhance catalytic activity. Besides the direct acquisition of genetic material from resistant bacterial species, the maintenance of spare copies of “ready-to-optimize” resistance genes in the genome of *A. baumannii,* perhaps selected by exposure to subinhibitory levels of the drug in the environment, might also play a role in its rapid adaptation to new derivatives of the major antibiotic classes.

## Materials and Methods

### *A. baumannii* strains.

Strains AYE and SDF were identified as *A. baumannii* using both phenotypic (API 20NE system; BioMerieux, Marcy l'Etoile, France) and genotypic methods [8,10]. Both strains were grown on trypticase-soy agar (BioMerieux).

### Sequencing.

Genomic DNA of AYE and SDF strains was mechanically sheared and fragments of 6 kb and 14 kb were cloned into two plasmid vectors, pNAV (A) and pCNS (B) (pcDNA2.1- and pSU18-derived, respectively). Plasmid DNAs were purified and end-sequenced by dye-terminator chemistry on ABI3730 sequencers (Applied Biosystems, Foster City, California, United States). The PHRED/PHRAP/CONSED software package was used for sequence assemblies. Gap closure and quality assessment were made for AbaR1 and AbaG1 islands.

### Annotation.

Initially, a preliminary set of putative ORFs of 300 nucleotides or more of both shotgun genome sequences was obtained. These included a proportion of hypothetical ORFs. The ORF nomenclature used in this study refers to this initial step. Then, the coding potential of ORFs was evaluated using the SelfID program, and systematic sequence similarity search of ORFs was conducted against the NCBI nonredundant protein database using the gapped BLASTP program. Gene identities were obtained using a reciprocal best match strategy. ORFs with neither coding potential nor similarity were no longer considered. Tentative ORF functions were assigned on the basis of sequence similarity against protein sequence databases (KEGG, COG, SWISSPROT, and the nonredundant protein database) and domain/motif databases (Pfam and PROSITE). Orthologous versus paralogous relationships were identified with multiple sequence alignments and neighbor-joining trees constructed by ClustalW. The functional classification was based on the scheme provided by the *Acinetobacter* spp. ADP1 genome project [32] as well as that provided by KEGG.

### Sources of software and databases.

The PHRED/PHRAP/CONSED software package is available at http://www.phrap.com. The SelfID program is available at http://igs-server.cnrs-mrs.fr/~audic/selfid.tgz. The gapped BLAST program is available at http://www.ncbi.nlm.nih.gov/BLAST. KEGG is available at http://www.genome.jp/kegg. COG is available at http://www.ncbi.nlm.nih.gov/COG. SWISSPROT is available at http://www.expasy.org/sprot. Pfam is available at http://pfam.wustl.edu. PROSITE is available at http://www.expasy.org/prosite.

## Supporting Information

Protocol S1 Molecular Detection of Genomic Islands in Clinical Isolates of *A*. *baumannii*
(25 KB DOC)

### Accession Numbers

The EMBL (http://www.ebi.ac.uk/embl) accession numbers for the resistance islands in the two *A. baumannii* strains are, for AYE, AbaR1 (CT025832); and for SDF, AbaG1 (CT025833). Other genes discussed, also in the EMBL database, are *ade*A (CT025811), *ade*B (CT025812), *ade*C (CT025813), *ade*I (CT025787), *ade*J (CT025788), *ade*K (CT025790), *ade*R (CT025814), *ade*S (CT025815), aminoglycoside adenylyltransferase (CT025824), *amp*C (CT025798), a putative β-lactamase (CT025947), *gyr*A (CT025946), *par*C (CT025948), *tet*A (AM086635). The GenBank (http://www.ncbi.nlm.nih.gov) accession number of *^bla^*OXA-69 is AY859527.

## Abbreviations

MDR: multidrug resistance (-resistant)
ORF: open reading frame
