# Supplementary material for: Comparative Genomics of Multidrug Resistance in Acinetobacter baumannii
Source: PLoS Genet. 2006 Jan 13;2(1):e7. doi: 10.1371/journal.pgen.0020007 (PMC1326220; doi:10.1371/journal.pgen.0020007)
Supplement: Supplementary file 1 [file pgen.0020007.sd001.doc]

**Protocol S1.**

**Molecular detection of genomic islands in clinical isolates of *A*. *baumannii*.**

We investigated the presence of an island in 22 clinical isolates with variable antimicrobial susceptibility profiles using a PCR targeting a 253-bp fragment of the ATPase gene overlapping the insertion site with the primers 42-156F (5’-ATTGATGTACCRCCATTA-3’) and 46-358R (5’-GTGAACCATCTTTGGGCA-3’). Of the 22 isolates, six had an extended-spectrum beta-lactamase (ESBL) phenotype (resistance to all beta-lactams except imipenem in four, and except imipenem and piperacillin-tazobactam and ticarcillin-clavulanate in two), including six which were also resistant to cotrimoxazole, three to fluoroquinolones, two to aminoglycosides, three to rifampin, and three to chloramphenicol; five isolates had a penicillinase phenotype (resistance to amoxicillin, ticarcillin and piperacillin), including five which were also resistant to cotrimoxazole, three to rifampin, three to chloramphenicol, and one to fluoroquinolones and aminoglycosides; four isolates were resistant to all beta-lactams except imipenem but did not have an ESBL phenotype, including four which were resistant to cotrimoxazole, two to chloramphenicol, and two to rifampin; and seven isolates were susceptible to all beta-lactams except penicillin G, including three which were resistant to cotrimoxazole, three to rifampin, two to chloramphenicol, and two to fluoroquinolones. In 17/22 strains, a PCR product of the expected size was obtained and the resulting sequences showed the ATPase gene to be free of insert. In the remaining five strains, no amplification could be obtained, thus suggesting the presence of a large-size insert. These five isolates included three isolates having an ESBL phenotype, one isolate resistant to all beta-lactams except imipenem without ESBL phenotype, and one isolate susceptible to all beta-lactams except penicillin G, and resistant to cotrimoxazole and rifampin.

*Acinetobacter baumannii* strains AYE and SDF were deposited in the strain collection of the Unité des Rickettsies and are available upon request.
